# Lightweight magnesium nanocomposites: electrical conductivity of liquid magnesium doped by CoPd nanoparticles

**DOI:** 10.1007/s13204-018-0789-6

**Published:** 2018-04-26

**Authors:** Andriy Yakymovych, Adam Slabon, Yuriy Plevachuk, Vasyl Sklyarchuk, Bohdan Sokoliuk

**Affiliations:** 1 Department of Inorganic Chemistry – Functional Materials, Faculty of Chemistry, University of Vienna, Althanstr. 14, 1090 Vienna, Austria; 2 Department of Metal Physics, Ivan Franko National University of Lviv, Kyrylo i Mephodiy str. 8, Lviv, 79005 Ukraine; 3 Institute of Inorganic Chemistry, RWTH Aachen University, Landoltweg 1, 52056 Aachen, Germany

**Keywords:** Mg-based alloys, CoPd nanoparticles, Nanocomposites, Electrical conductivity, Microstructural analysis

## Abstract

The effect of monodisperse bimetallic CoPd NP admixtures on the electrical conductivity of liquid magnesium was studied. Temperature dependence of the electrical conductivity of liquid Mg_98_(CoPd)_2_, Mg_96_(CoPd)_4_, and Mg_92_(CoPd)_8_ alloys was measured in a wide temperature range above the melting point by a four-point method. It was shown that the addition of even small amount of CoPd nanoparticles to liquid Mg has a significant effect on the electrical properties of the melts obtained.

**Electronic supplementary material:**

The online version of this article (10.1007/s13204-018-0789-6) contains supplementary material, which is available to authorized users.

## Introduction

Due to light weight combined with good mechanical properties, such as the high specific strength and ductility, magnesium alloys are widely used in the automotive and aerospace industries. For instance, AZ31 as one of the most used Mg-based alloys is employed in the automotive industry since the last 4 decades (Bettles and Barnett 2012). The special feature of these Mg-based alloys is that they have a stringy texture in the deformation direction, which increases the tensile proof strength. However, their chemical composition is complicated and includes at least seven pure metals as alloying elements.

Among various methods to improve microstructure and enhance the physical and mechanical properties of different materials, a minor addition of nano-sized particles is very popular at the moment. For instance, there are a number of reports describing additions of metal or ceramic nanoparticles (NPs) to commercial lead-free solders (Yakymovych et al. 2017, 2016; Yakymoyych et al. 2016). Investigations of aluminum- or magnesium-based matrix composites with ceramic nanoinclusions were focused mostly on microstructural analysis and mechanical properties of such nanocomposite alloys (Jiang and Wang 2015; Casati and Vedani 2014; Chen et al. 2015). At the same time, there are no literature reports devoted to properties of aluminum- or magnesium-based matrix composites with nanoscopic metal inclusions. However, a possible industrial application of nanocomposite metal materials could raise many questions related to the thermodynamic stability of nanoscopic inclusions in the bulk, especially if using chemically reactive metal NPs. Chemical reactions could lead to dissolution of NPs in the matrix or formation of new phases. These structural transformations could be reflected in changes of physical and mechanical properties, and should be examined in detail. Therefore, investigations of the microstructure and characteristics of nanocomposite materials with respect to the temperature and pressure would provide essential information for the development of new commercial materials and optimization of their production processes.

In the present work, the temperature dependence of the electrical conductivity of the liquid Mg with minor addition of nanoscopic CoPd particles was investigated. Nanocomposite samples were prepared from Mg powder and monodisperse bimetallic CoPd NPs. The analysis of the heating and cooling curves of the electrical conductivity provides important information regarding both the changes of electrical properties and the dissolution process of CoPd NPs in the liquid Mg matrix during heating. To the best of our knowledge, there are no literature data related to the structure and thermophysical properties of Mg alloys with nanoscopic metal inclusions in the liquid state after melting. At this point, the present study will provide new experimental data which can be very useful for comparison with model predicted values.

## Experimental

### Synthesis of CoPd NPs

Oleylamine (80–90%, Sigma-Aldrich) was distilled under vacuum twice before the reaction. CoPd NPs were synthesized by a modified synthetic procedure which was reported by Mazumder et al. (2012). 20 mL of oleylamine were loaded in a three-neck flask and the solution degassed at 403 K for 2 h under the flow of argon. After cooling down to the room temperature, 0.30 mmol of palladium bromide (PdBr_2_, 99.0 wt%, Sigma-Aldrich) and 0.20 mmol of cobalt acetate (Co(ac)_2_, 99.995 wt%, Sigma-Aldrich) were added to the solution. The flask was enclosed and quickly heated under the argon flow to 333 K. After reaching this temperature, 0.5 mL of trioctylphosphine (TOP, 97 wt%, Sigma-Aldrich) was added to the solution. The temperature was increased to 533 K and heated for 2 h. Subsequently, the mixture was cooled down to 323 K and the NPs were precipitated with 25 mL of ethanol (absolute, 99.95% Chemsolute GmbH). The CoPd NPs were centrifuged at 12.000 rpm for 20 min, redispersed in hexane and washed twice with 25 mL of ethanol.

### Preparation of nanocomposite samples

The composite Mg_100−x_(nanoCoPd)_x_ alloys were prepared by mixing Mg powder (∼25 mesh; 99.8%; Alfa Aesar, Germany) with 2.0, 4.0, and 8.0 at.% of synthesized CoPd NPs. Mechanical dispersion of the NPs in the Mg powder was achieved by mechanically mixing, maintaining a frequency of 700 rpm (11.7 s^−1^) at room temperature for approximately 30 min using a Retsch mixer (Retsch MM301). After that, the Mg_100−x_(nanoCoPd)_x_ powders were pressed into cylindrical form (about 3 mm diameter and about 10 mm height).

### Investigation of the electrical conductivity

The electrical conductivity measurements were carried out by the 4-point method in an argon atmosphere. Graphite electrodes for current and potential measurements were placed in the wall of the vertical cylindrical boron nitride (BN) ceramic measuring cell along its vertical axis. The potential electrodes were provided with thermocouples for temperature measurements. Single thermoelectrodes of these thermocouples were used for electrical conductivity determination. The melt temperature was determined by WRe-5/20 thermocouples located in close contact with the liquid. Further details of this method and its experimental realization have been described elsewhere (Plevachuk and Sklyarchuk 2001). Each sample was inserted into the cell directly inside a high-pressure vessel. Thus, the actual sample composition was accurate within a tolerance of 0.02 wt%. The resultant uncertainty of the electrical conductivity measurements is about 2%.

### Microstructural analysis of samples

The phase composition of the samples after electrical conductivity measurements was analyzed by X-ray diffraction (XRD) and scanning electron microscopy (SEM) methods. Powder XRD measurements were done on a Bruker D8 diffractometer at ambient temperature using Ni filtered Cu K_α_ radiation (accelerating voltage 40 kV, electron current 40 mA). The diffractometer operates in the *θ*/2*θ* mode. The powder was fixed with petroleum jelly on a single-crystal silicon sample carrier which was rotated during the measurement. The detection unit was a Lynxeye strip detector. Indexing of the phases was supported by the Inorganic Crystal Structural Database (ICSD). Rietveld refinement of the XRD patterns was done with the Topas3® software provided by Bruker AXS.

The scanning electron microscope Zeiss Supra 55 VP was used for metallographic investigations. The excitation energy of the electron beam was 15–20 kV; backscattered electrons (BSE) were detected to visualize the surfaces of the samples. Pure Co was also used for energy calibration of the EDX detector signal. An acceleration voltage of 20 kV was applied. The chemical compositions obtained from EDX differ from the corresponding average value up to ± 1 at.%.

## Results and discussion

### Structural characterization of CoPd NPs

**Fig. 1:**
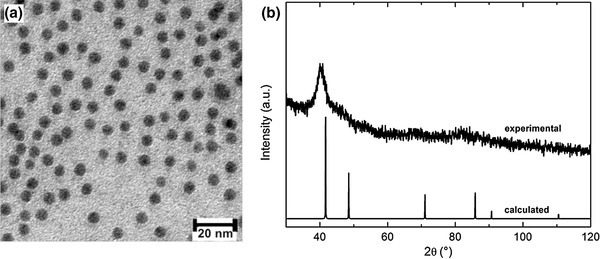
TEM image (**a**) with corresponding XRD patterns (**b**) of self-made CoPd NPs

Figure 1a shows a TEM image of synthesized CoPd NPs. The monodisperse and spherical NPs display a mean average particle size of 5.4 ± 0.6 nm (based on 150 counted entities). This mean value is slightly larger by 0.1 nm in comparison to the previous work by Davi et al. (2017). The corresponding powder XRD patterns exhibit broad reflection peaks of a face-centered cubic (fcc) structure (Fig. 1b). Its bimetallic composition of Co_x_Pd_1−x_ (x = 0.4) was confirmed by means of atomic absorption spectroscopy (Davi et al. 2017).

### Electrical conductivity of magnesium-based nanocomposites

According to former investigations, the electrical resistivity in all alkaline earth metals increases abruptly at the melting point and reveals weakly negative temperature dependence in the liquid phase (Chi 1979). These sudden changes are connected with a greater disorder of the liquid state and a disappearance of any definite crystal structure.

**Fig. 2:**
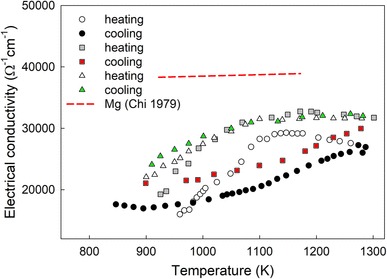
Temperature dependence of the electrical conductivity of liquid Mg (red dashed line—Chi 1979) and liquid Mg–(CoPd) alloys [opened triangle, green filled triangle—Mg_98_(CoPd)_2_; grey filled square, red filled square—Mg_96_(CoPd)_4_; opened circle, filled circle—Mg_92_(CoPd)_8_]

Although the electrical resistivity of magnesium in the solid state was studied repeatedly, only several data sets on the electrical resistivity in the liquid state had been published (see Chi 1979 and references therein). A range of the reported electrical resistivity data are rather large, which can be caused by experimental difficulties due to a high melting temperature of Mg (922 K) as well as a very high vapor pressure (1600 Pa at 1000 K). Furthermore, both the positive and negative temperature dependence of the electrical resistivity were found. Comparison with the electrical resistivity data of other alkaline earth elements in the liquid state suggests that the electrical resistivity of liquid Mg should have relative weak negative temperature dependence. Based on the available experimental data, a linear equation for description of the electrical resistivity in the temperature range between 922 and 1171 K has been proposed in Chi (1979). Figure 2 presents the temperature dependence of the electrical conductivity *σ* (*T*) for liquid Mg, converted from the resistivity data.

Temperature dependence of the electrical conductivity for liquid Mg with nano-sized CoPd admixtures is more complicated (see Fig. 2). It should be mentioned that although the NPs were monodisperse entities, one cannot exclude partial agglomeration of CoPd NPs during the preparation of the sample.

According to experimental data, the electrical conductivity behavior of liquid Mg with CoPd NPs is typical for intermetallic semiconducting melts (Hlil et al. 2010; Stadnyk et al. 2010). Beginning from the melting point, the electrical conductivity of the investigated Mg–(nanoCoPd) samples with 2, 4, and 8 at.% of nanoinclusions increases upon heating similar to the conductivity of liquid Mg.

The SEM–EDX and XRD analysis revealed formation of a phase Mg_6_Pd with a predominant homopolar type of chemical bonds. Obviously, a presence of such homopolar bonds in the melt leads to manifestation of the semiconducting nature of the electrical conductivity change. The initial increase of the electrical conductivity after melting indicates a release of a certain number of free electrons. However, further heating in the liquid state leads to an increase in electrical conductivity, and subsequently to its reduction, which is unusual for metals. Apparently, this is due to the thermal dissociation of metal compounds, formed in the Mg–CoPd alloys. This is most pronounced for the Mg_92_(CoPd)_8_ alloy and, to a lesser extent, for Mg_98_(CoPd)_2_.

The cooling *σ*(*T*) curves, convex for Mg_98_(CoPd)_2_ and concave for Mg_98_(CoPd)_2_ and Mg_96_(CoPd)_4_ alloys, are more smooth and can be described by the second-order polynomial:

1 $$\sigma (T)={\sigma _0}+a\left( {T - {T_{\text{L}}}} \right)+b{\left( {T - {T_{\text{L}}}} \right)^2},$$

where *T*_L_ is a liquidus temperature determined from electrical conductivity measurements upon cooling, *σ*_0_ is the electrical conductivity value at *T*_L_. The fitting parameters used in the Eq. (1) are presented in Table 1.

**Table 1:** Parameters *T*_L_, *σ*_0_, *a*, and *b* used in Eq. (1)

Composition (at.%)	*T*_L_ (K)	*σ*_0_ (Ohm^−1^ m^−1^)	*a* (Ohm^−1^ m^−1^ K^−1^)	*b* (Ohm^−1^ m^−1^ K^−2^)
Mg_98_(CoPd)_2_	910	24,575	49.0146	− 0.0786
Mg_96_(CoPd)_4_	897	20,993	3.9166	0.0529
Mg_92_(CoPd)_8_	846	17,213	− 2.1425	0.0611

The electrical conductivity of the investigated Mg^+^(CoPd) alloys is lower than conductivity of pure Mg, while the smallest values were obtained for the sample with the greatest amount of CoPd admixtures. Such behavior generally agrees with the previous studies of systems, in which even small additions of transition metals to a basic metal matrix affected considerably the electron structure of the system and the electronic properties (Plevachuk et al. 2006; Sklyarchuk and Plevachuk 2003; Plevachuk and; Sklyarchuk et al. 2008). It was shown that small additions (within a few percent) of 3*d* transition metals can affect considerably the electronic structure of the systems and their electronic properties. In similar alloys, a gradual increase of the transition metal content is accompanied by a decrease of the number of conduction electrons. In other words, the condition *K*_p_ ≈ 2*k*_f_ is valid, leading to a conductivity minimum and to a transition to the positive d*σ*/d*T* values. Here, *K*_p_ is a wave number at the structure factor maximum, *k*_f_ is the Fermi wave number (Gilman and Leamy 1978). Thus, according to the Ziman theory (Ziman et al. 1969), the electrical conductivity decreases from Mg to Mg_92_(CoPd)_8_. As shown in Fig. 2, this decrease is also accompanied by a change in the shape of the curves *σ*(*T*).

According to the model of substitution (Ziman et al. 1969), the electrical conductivity of a binary melt is expressed as follows:

2 $$\frac{1}{\sigma }=\frac{1}{{{\sigma _1}}}+\frac{1}{{{\sigma _2}}},$$

3 $$\frac{1}{{{\sigma _1}}}=\frac{{3\pi \Omega }}{{\hbar {e^2}v_{{\text{F}}}^{2}}}\int\limits_{0}^{1} {\left[ {\left( {1 - {c_1}} \right)a(K)\left| {{w_1}(K)\left| {^{2}} \right.+{c_1}a(K)\left| {{w_2}(K)\left| {^{2}} \right.} \right.} \right.} \right]} 4{\left( {\frac{K}{{2{k_{\text{F}}}}}} \right)^3}d\left( {\frac{K}{{2{k_{\text{F}}}}}} \right),$$

4 $$\frac{1}{{{\sigma _2}}}=\frac{{3\pi \Omega }}{{\hbar {e^2}v_{{\text{F}}}^{2}}}\int\limits_{0}^{1} {\left\{ {{c_1}\left( {1 - {c_1}} \right)\left[ {1 - a(K)} \right]{{\left[ {{w_2}(K) - {w_1}(K)} \right]}^2}} \right\}} 4{\left( {\frac{K}{{2{k_{\text{F}}}}}} \right)^3}d\left( {\frac{K}{{2{k_{\text{F}}}}}} \right),$$

where Ω is the atomic volume, *c*_1_ is a content of one of the components, and *w*_1_*(K)* and *w*_2_*(K)* are the pseudopotentials of the components 1 and 2, respectively. The *σ*_*1*_ value should change roughly linearly with composition within the electrical conductivities of the two components. The magnitude of *σ*_2_ includes the composition dependent quadratic term into the conductivity, according to the Nordheim rule. A general view of the melt conductivity isotherm will be either linear or nonlinear, depending on which term in Eq. (2) makes a predominant contribution.

If the additional impurity is a 3*d*(4*d*)-, transition metal like Co, Pd, or both in our case, the nearly free electron approximation is not suitable. The electron scattering by impurity transition atoms falls mainly into the *d*-orbital, which is rather localized but in resonance with the conduction band. Thus, in the case of a transition metal, a large part of scattering is due not only to *s*- and *p*-waves but also to *d*-waves. This is connected with the fact that the level of the localized *d*-electrons in metals seems to exist in the same region of energy as the conduction band and hence the resonance between the *d*-level and the conduction band must give rise to a virtual bond state (Plevachuk et al. 2006; Friedel 1956). Therefore, the method of partial waves is preferred. An increase of the CoPd NPs in the melt leads to an increase of the virtual bond states, which is reflected by the decrease of the absolute value of the electrical conductivity (Fig. 2).

Thus, the electrical conductivity behavior reflects complicated mechanisms of simultaneous formation and dissociation of metallic compounds, indicating that molten metallic alloys can undergo a number of structural transformations from the initial microheterogeneous state immediately after melting to the true solution state. A temperature range of these transformations can be rather wide, as in the case of the investigated liquid Mg_98_(CoPd)_2_, Mg_96_(CoPd)_4_, and Mg_92_(CoPd)_8_ alloys, which reveal the electrical conductivity behavior typical for intermetallic semiconductors. At the same time, the high absolute values of the electrical conductivity are higher than for intermetallic semiconductors and correspond to the metal alloys.

### Microstructural analysis of samples

**Fig. 3:**
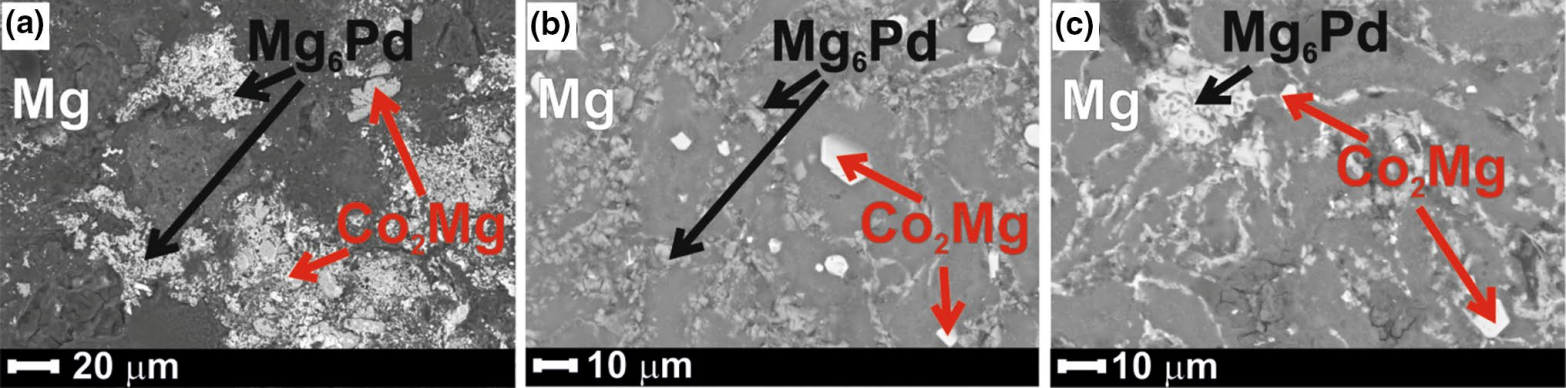
BET micrographs of Mg_100−x_(CoPd)_x_ (**a** Mg_98_(CoPd)_2_; **b** Mg_96_(CoPd)_4_; **c** Mg_92_(CoPd)_8_) samples after measurements of the electrical conductivity

In order to prove that CoPd NPs added to Mg had dissolved completely during the measurements of the electrical conductivity, the alloys were investigated by means of SEM–EDX and powder XRD measurements after rapid cooling down in the apparatus. The results of phase analyses and BSE images can be found in Table 2 and Fig. 3, respectively.

**Table 2:** Phase composition of cooled Mg_100−x_(CoPd)_x_ samples after measurements of the electrical conductivity (results of SEM–EDX)

Sample	Mg (at.%)		Co–Mg phase (at.%)			Mg–Pd phase (at.%)		
				Co	Mg		Mg	Pd
Mg_98_(CoPd)_2_	α-Mg	100	Co_2_Mg	65	35	Mg_6_Pd	90	10
Mg_96_(CoPd)_4_	α-Mg	100	Co_2_Mg	62	38	Mg_6_Pd	87	13
Mg_92_(CoPd)_8_	α-Mg	100	Co_2_Mg	66	34	Mg_6_Pd	91	9

**Fig. 4:**
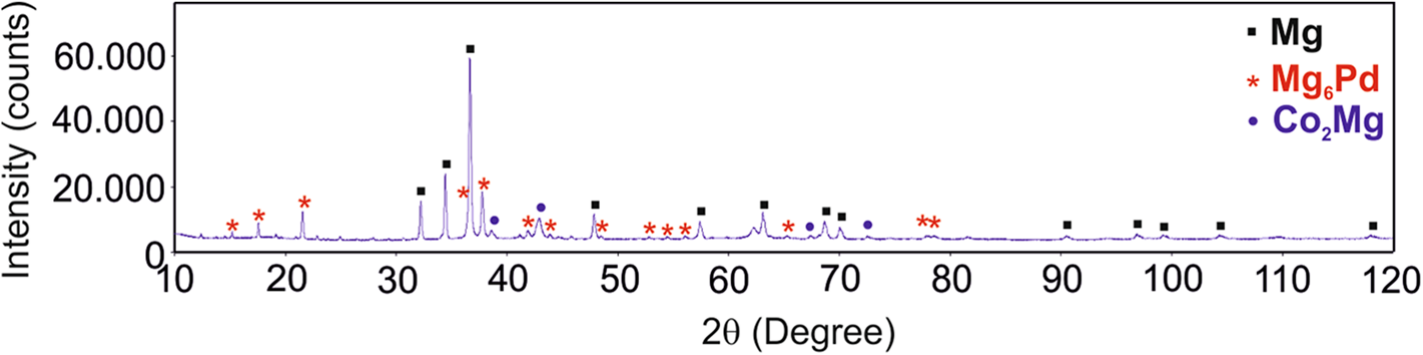
XRD pattern of the Mg_92_(CoPd)_8_ alloy

No residual pure Co or Pd was found in the investigated samples. Therefore, it is suggested that the nanoscopic CoPd inclusions were totally dissolved in liquid Mg matrix during the measurements of the electrical conductivity. According to the phase diagram of Mg–Pd system (Nayeb-Hashemi and Clark 1985; Makongo et al. 2006), there are several Mg-rich Mg–Pd phases, while Mg_6_Pd has the highest Mg content. Instead, Co_2_Mg is the only one thermodynamically stable phase in Co–Mg (Massalski and Okamoto 1990). Due to the small additions of Co and Pd into pure Mg (maximum amount of each element equals 4 at.%), it is practically very complicated to distinguish the corresponding reflection peaks. However, there is a good agreement between the results of the SEM–EDX analysis and XRD. For instance, as shown in Fig. 4, the peaks corresponded to pure Mg, Co_2_Mg, and Mg_6_Pd phases could be indicated in the XRD pattern of the Mg_92_(CoPd)_8_ sample.

## Conclusion

The influence of nanoscopic bimetallic CoPd admixtures on the electrical conductivity of liquid magnesium was studied. Temperature dependence of the electrical conductivity of liquid Mg_98_(CoPd)_2_, Mg_96_(CoPd)_4_ and Mg_92_(CoPd)_8_ alloys was measured in a wide temperature range above the melting point by a four-point method. It was revealed that the electrical conductivity behavior reflects the complex mechanisms of simultaneous formation and dissociation of metallic compounds. The results indicate that molten metallic alloys can undergo a number of structural transformations from the initial microheterogeneous state immediately after melting to the real solution state. The temperature range of these transformation can be rather wide, as in the case of liquid Mg_98_(CoPd)_2_, Mg_96_(CoPd)_4_, and Mg_92_(CoPd)_8_ alloys, which reveal typical for intermetallic semiconductors electrical conductivity behavior. At the same time, the high absolute values of electrical conductivity are typical for metal alloys. Thus, our study proved that addition of even small amount of NPs can have a significant effect on electrical properties of Mg matrix in the liquid state. Additional studies of possible similar effect of NPs in the solid state are foreseen.

## Electronic supplementary material

Below is the link to the electronic supplementary material.

Supplementary material 1 (PNG 287 KB)
