# Supplementary figures and images for: Lightweight magnesium nanocomposites: electrical conductivity of liquid magnesium doped by CoPd nanoparticles
Source: Appl Nanosci. 2018 Apr 26;9(5):1119–25. doi: 10.1007/s13204-018-0789-6 (PMC6661030; doi:10.1007/s13204-018-0789-6)

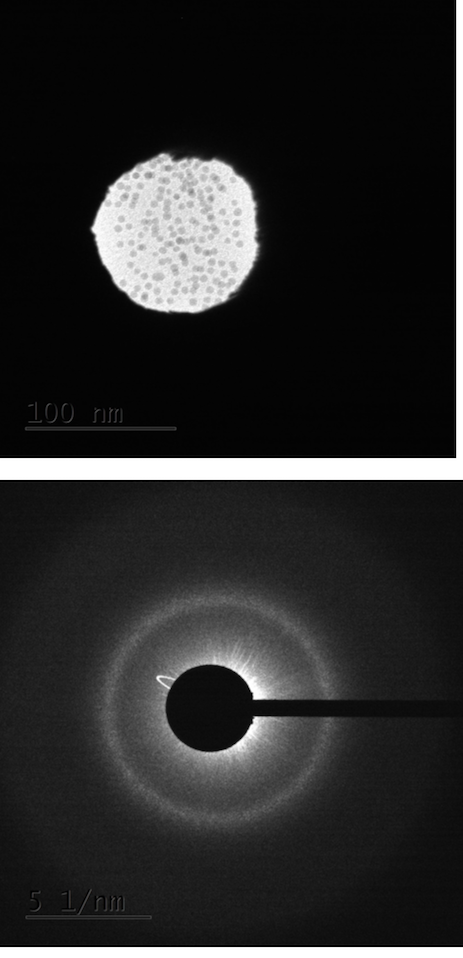

Supplement: Supplementary file 1 — Supplementary material 1 (PNG 287 KB) [file 13204_2018_789_MOESM1_ESM.png]
